# Event and Comment

**Published:** 1919-01

**Authors:** 


### DENTAL REGISTER

Vol. LXXIII JANUARY, 1919 No. 1

### EVENT AND COMMENT

**Dental Review Ceases Publication** After thirty-two years of faithful service this most excellent dental journal ceases publication to the great regret of all lovers of the profession who have received inspiration and instruction of incalculable value through its continuous efforts to present the professional activities of the dental world. The *Review* was started by an association of the live workers of the profession to present and preserve the activities of the profession in the vicinity of Chicago, which at that time was rapidly becoming the center of professional activity in this country, if not of the world. How much this journal has had to do with making the middle west what it is today it would be difficult to determine, but certainly it has been one of the greatest factors. Dr. A. W. Harlan, one of the most tireless workers our profession ever knew was its first editor, with J. W. Wassal, Louis Ottoby, J. G. Reid and L. C. Davis, as associates. With such a staff this journal's character was assured from the start, and such men as have become associated with its conduct since have continually made it one of the leading and dependable factors in all our associated efforts.

The publishers announce its discontinuance because of the expense involved in its publication and because of the new postal laws, etc. It seems incredible that the profession of dentistry should be willing to permit as important a force for good as this journal has proven itself to be, to be given up for any such reasons. The announcement is made in this connection that the *Journal of the National Dental Association* will hereafter publish the material formerly given to the profession through the *Dental Review.* Possibly this may be done to an extent, but we are somewhat doubtful if that journal will be able to carry all the material contributed formerly through the *Review* in addition to the work it has already assumed. We are quite sure it can not from the very nature of the case take the place that the *Review* has made for itself in the hearts of its readers, any more than a letter, however interesting, from *someone, somewhere* in France, is as comforting to us as a letter from our own flesh and blood who is doing his best *somewhere* in the great struggle for freedom and liberty. We can not think of this proposition other than as more or less fatal to the development of the individuals composing the great majority of our profession. Our literature of the future seems tending toward a class of writers on mere technical details and questionable ethical practices for publication in trade journals, which are increasing in numbers and will necessarily reach the profession more generally and be read perhaps more carefully than that other class of journals which endeavor to carry only the better literature, and the results of scientific research, which the large majority do not care to read and can not appreciate. We surely need the *Journal of the National Dental Association,* and there may be a place of value in our development for the mere trade publication, if decently conducted; but there certainly is an important place for a journal, such as the *Dental Review* has so admirably exemplified, and we can not but deplore the fact that it has quit the cause at so important a period of our development. As we were not consulted we shall have to be resigned, but can't help protesting. Let us hope that some new order will appear to keep the old fires burning while our hearts are yearning for the tried and true which shall abide as a basis of future development.

**C. N. Johnson's Editorial Retirement**

The *Dental Review* ceased to exist with the December, 1918, issue, and Dr. Johnson is out of a job. If this sentence contained the whole truth and the consequences were ordinary, we should congratulate our confrere that he is released from the cares of a most exacting occupation, and we should have a new cause for envying one whom we so often have considered specially favored, not only providentially, but because of his remarkable abilities to carry the larger tasks so creditably. We regret our incapacity to put in form the thoughts that have passed through our minds since learning of the passing of the *Dental Review* and the loss of the monthly visit with an esteemed friend and confrere. Neither have we the space here for such an effort, but we can't refrain from a brief comment in which we shall try to express what we feel must be the sentiment of all who have known Dr. Johnson in this particular field of professional work.

Dr. Johnson began his editorial work on the *Review* as associate editor, with Dr. A. W. Harlan as his chief, in 1890, and assumed full editorial charge with the January issue, 1894, and has continued faithfully to date in forwarding the best ideals and activities of the profession. Characteristic of the man in his salutatory editorial he promises to give his editorial kite all the string he possesses and asks for the co-operation of the profession. As we review his work, both he and the profession have made good, and if we mistake not, it has been very largely Dr. Johnson's ability to secure the confidence and affection of his constituents that has made the *Review* the power for good it has always exerted and which has kept him faithfully on his job all these years. In fact it has not been a *job* at all with him, it has been an unceasing devotion to a cause which has seemed to him deserving of all that was most noble and desirable in life. He seems to have imbibed the spirit of Hiram Goff, who said "he cobbled shoes by the grace of God," so Dr. Johnson became the powerful factor in the dental world that he is, because he considered his call to serve in this particular sphere divine rather than self sought.

In his valedictory editorial he expresses his regret that he has occupied so much space with his own writings—"sixty-one of his own papers and 507 editorials"—no one but an editor can appreciate the meaning of this large contribution to one subject. It makes one weary to think of the labor and time taken away from the other things he might have done. Did he ever play, or be amused, or make money for himself? And yet he found time for all of these things in a reasonable way, as we all know. Besides this, he wrote on other topics than dentistry. We have a little booklet of charming verses on some of the commonplace events of life which he thought out and recorded as an idealist. It is the fact that he has such an absorbing interest in the best things in life that we regret most his retirement from the editorial work for which he is so admirably endowed and inclined. It has taken more courage than we suppose for Dr. Johnson to write the charming notes on travel and life incidents and print them in the editorial pages of a formal professional journal, but he has done it so discreetly that no one has been bold enough to criticise. After all, how much more in keeping with real professionalism than the cheap wit that is so prevalent in some of our journals, or even the questionable character of many of the technical articles sometimes encountered.

Although the *Review* has ceased publication and Dr. Johnson will not edit, he has promised not to quit writing, which is some compensation. We do not know what he will write about, but we are sure he will write, he can't help it. There is so much that ought to be said, especially by men who have the best interests of our profession at heart, and who, because of their larger contacts with dental affairs, have accumulated wisdom and judgment which will give weight to their words.

We wish our friend all the happiness to be had by the freedom from care that he has so fully earned, and we hope now that he may have all choice in selecting his topics and all the time he desires in which to compose whatever his hands find time to do and his mind and heart to conceive.

**Plate Prosthesis** We are giving much of our space in this issue to plate prosthesis. We feel confident that a careful perusal of the compiled articles will not only serve to give the best up-to-date material on this most interesting topic, but may, we hope, create a renewed interest if not some enthusiasm for better endeavor along this line. Our summary will help to give a good idea of what is being done by the best workers and we earnestly commend the original publishings with their illustrations for more careful reading.

